# Arterial Pulsatility Index in Acute Myocardial Infarction‐Related Cardiogenic Shock: Clinical Characteristics and Association With Outcomes

**DOI:** 10.1161/JAHA.125.047854

**Published:** 2026-08-20

**Authors:** Jonas Sundermeyer, Song Li, Van‐Khue Ton, Rachna Kataria, Elric Zweck, A. Reshad Garan, Manreet K. Kanwar, Jaime Hernandez‐Montfort, Shashank S. Sinha, Jacob Abraham, Kevin J. John, Paavni Sangal, Claudius Mahr, Peter S. Natov, M. Imran Aslam, Daniel Burkhoff, CSWG Academic Research Consortium, Navin K. Kapur

**Affiliations:** ^1^ Department of Cardiology University Heart and Vascular Center Hamburg, University Medical Center Hamburg‐Eppendorf Hamburg Germany; ^2^ The Cardiovascular Center, Tufts Medical Center Boston MA USA; ^3^ German Center for Cardiovascular Research (DZHK), Partner Site Hamburg/Kiel/Lübeck Hamburg Germany; ^4^ Institute for Advanced Cardiac Care Medical City Healthcare Dallas TX USA; ^5^ Massachusetts General Hospital Boston MA USA; ^6^ Brown University Health Cardiovascular Institute Providence RI USA; ^7^ Department of Cardiology, Pulmonology, and Vascular Medicine Medical Faculty and University Hospital Düsseldorf, Heinrich‐Heine‐University Düsseldorf Germany; ^8^ Beth Israel Deaconess Medical Center Boston MA USA; ^9^ University of Chicago Chicago IL USA; ^10^ Baylor Scott & White Health Advanced Heart Failure Program Clinic Temple TX USA; ^11^ Inova Heart and Vascular Institute Inova Fairfax Campus Falls Church VA USA; ^12^ Center for Cardiovascular Analytics, Research, & Data Science (CARDS) Providence St. Joseph Research Network Portland OR USA; ^13^ Cardiovascular Research Foundation New York NY USA

**Keywords:** acute myocardial infarction, aortic pulsatility index, arterial pulsatility index, cardiogenic shock, hemodynamics, outcome, Myocardial Infarction, Heart Failure

## Abstract

**Background:**

The arterial pulsatility index (API) is a promising hemodynamic surrogate of left ventricular function, but its prognostic value in acute myocardial infarction‐related cardiogenic shock (AMI‐CS) remains unclear. This study evaluated associations between API, a modified API (mAPI), clinical characteristics, and in‐hospital outcomes in AMI‐CS.

**Methods:**

Patients with AMI‐CS in the multicenter CSWG (Cardiogenic Shock Working Group) registry with available API or mAPI were analyzed. API was calculated as systemic arterial pulse pressure/pulmonary capillary wedge pressure; mAPI used pulmonary artery diastolic pressure instead of wedge pressure. Logistic/linear regression, spline modeling, and receiver operating characteristic curve analysis were used to assess associations with clinical characteristics and in‐hospital outcomes.

**Results:**

Among 487 patients with AMI‐CS, median API and mAPI were 2.1 (interquartile range [IQR], 1.3–3.2; n=139) and 1.7 (IQR, 1.0–2.8; n=487), respectively. API strongly correlated with mAPI (Pearson *r*=0.75, *P*<0.001). Native heart survival was lower in patients with low API (41.4% versus 59.4%, *P*=0.051) or mAPI (41.2% versus 59.1%, *P*<0.001). Lower API or mAPI correlated with reduced left ventricular ejection fraction and was associated with higher in‐hospital mortality (47.1% versus 24.6%; odds ratio [OR], 2.728 [95% CI, 1.341–5.705]; *P*=0.006; and 44.1% versus 32.2%; OR, 1.657 [95% CI, 1.147–2.402]; *P*=0.007). Every 0.5‐unit decrease in API or mAPI was associated with a 2.4% (β=0.024 [95% CI, 0.002–0.045]; *P*=0.030) and 1.4% (β=0.014 [95% CI, 0.002–0.026]; *P*=0.022) increase in in‐hospital mortality.

**Conclusions:**

In AMI‐CS, lower API and mAPI were associated with increased mortality. API and mAPI may facilitate early risk assessment and guide tailored treatment decisions in AMI‐CS.

## Graphical abstract

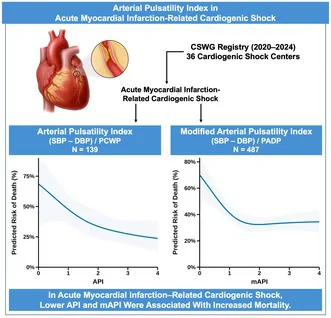

**Nonstandard Abbreviations and Acronyms**
API: arterial pulsatility index
CS: cardiogenic shock
CSWG: Cardiogenic Shock Working Group
mAPI: modified arterial pulsatility index
PADP: pulmonary artery diastolic pressure
SCAI: Society for Cardiovascular Angiography and Interventions
tMCS: temporary mechanical circulatory support

**Clinical Perspective**

**What Is New?**

- In this multicenter study, the lower arterial pulsatility index and modified arterial pulsatility index, measured early and before initiation of temporary mechanical circulatory support, were associated with treatment escalation, reduced cardiac recovery, and increased in‐hospital mortality in acute myocardial infarction‐related cardiogenic shock.

**What Are the Clinical Implications?**

- Establishing the arterial pulsatility index and modified arterial pulsatility index as hemodynamic markers of shock severity may enable future clinical studies to improve patient risk stratification and optimize tailored therapeutic decision‐making in acute myocardial infarction‐related cardiogenic shock.

Cardiogenic shock (CS) is a life‐threatening condition characterized by a significant reduction in cardiac output resulting in systemic hypoperfusion and potential multiorgan failure.^1^, ^2^, ^3^ Although evidence supports that early revascularization and temporary Impella CP support improve outcomes in selected acute myocardial infarction‐related CS (AMI‐CS) subsets, evidence for effective pharmacologic or device‐based treatment approaches across the broader CS population remains limited.^4^, ^5^, ^6^, ^7^, ^8^, ^9^, ^10^, ^11^ Despite optimal resources, including temporary mechanical circulatory support (tMCS) availability and an interdisciplinary shock team, determining timing, patient selection, and escalation criteria remains a clinical challenge.^1^, ^2^, ^3^, ^12^ In this context, pulmonary artery catheters (PACs) may additionally guide triage, vasoactive therapy, and tMCS management but are not commonly used worldwide during AMI‐CS.^13^, ^14^, ^15^, ^16^

The arterial pulsatility index (API), defined as the ratio of systemic pulse pressure to pulmonary capillary wedge pressure (PCWP), is an invasive hemodynamic metric that has been proposed as an index of left ventricular (LV) performance.^17^ However, measurement of PCWP is not a standard of care in many intensive care units around the world. Because pulmonary artery diastolic pressure (PADP) largely correlates with PCWP in patients with CS, a modified API (mAPI), based on PADP instead of PCWP, may represent a more generally available variant of this index with equivalent clinical meaning.^18^

In AMI‐CS, acutely impaired LV contractility and elevated filling pressures represent hemodynamic factors that contribute to circulatory failure.^19^ Accordingly, API and mAPI may serve as a simple, readily obtainable index for risk stratification and for guiding tailored treatment approaches in AMI‐CS. However, the association of API and mAPI with outcomes in AMI‐CS has not yet been evaluated in clinical settings.

This study aimed to evaluate the association between API and mAPI with clinical characteristics and in‐hospital outcomes in patients with AMI‐CS, using data from the large, contemporary, multicenter CSWG (Cardiogenic Shock Working Group) registry.

## METHODS

### Data Availability

The data that support the findings of this study are available from the corresponding author upon reasonable request.

### Data Source and Setting

The CSWG registry is an international, multicenter CS database, established and maintained by the CSWG academic research consortium, enrolling consecutive patients from currently 36 participating CS centers. The CSWG registry captures both prospective and retrospective standardized sets of data elements, including demographics, comorbidities, laboratory results, hemodynamic profiles, and drug‐ and device‐based treatments strategies, including different treatment approaches (conservatively or with tMCS) at baseline and at predefined time points over the first 72 hours of the onset of shock. At each site, CS diagnosis was adjudicated by the treating physicians and defined as sustained cardiac dysfunction meeting at least 1 of the following: systolic blood pressure <90 mm Hg for ≥30 minutes, need for vasoactive medications, cardiac index <2.2 L/min per m^2^ in the absence of hypovolemia, or initiation of tMCS for suspected CS. CS management, including selection of therapeutic interventions, was not standardized and was determined by local treating physicians according to site‐specific practice and guidelines at each participating center. CSWG principal investigators ensured data quality through case adjudication, centralized audits, and resolution of discrepancies with participating sites. Further details on eligibility criteria, data entry procedures, and registry conduct were predefined by the CSWG principal investigators and are described elsewhere.^13^, ^20^, ^21^

This analysis was conducted in accordance with the Declaration of Helsinki and received approval for patient enrollment from the institutional review boards or local ethics committees at each participating center. Written informed consent was waived for the retrospectively included patients in accordance with the approval of the local ethics committees.

### Index Eligibility Criteria and Definition of Study Groups

For the final analysis, patients with CS enrolled from 2020 to 2024 were included. Only cases classified as AMI‐CS, defined as CS caused by a primary diagnosis of ST‐segment–elevation myocardial infarction or non–ST‐segment–elevation myocardial infarction, were analyzed. Society for Cardiovascular Angiography and Interventions (SCAI) stages were assigned according to the CSWG‐SCAI definitions as described previously.^21^ Out‐of‐hospital cardiac arrest was not considered as an automatic criterion for classification as SCAI stage E.

The API was calculated as the ratio of systemic pulse pressure (systolic blood pressure minus diastolic blood pressure) to PCWP. The mAPI was calculated replacing PCWP with PADP. PCWP and PADP were obtained via PAC measurements.

For this analysis, only patients with available systolic and diastolic blood pressure and PCWP or PADP were included. Systolic and diastolic pressures were obtained from peripheral arterial measurements (invasive arterial line or noninvasive brachial blood pressure) at the time of PAC measurements. Central aortic pressures were not captured. Accordingly, the indices are referred to as the API (rather than aortic), reflecting reliance on clinically available, routinely measured pressures and broad applicability in a real‐world CS setting.

Variables for API or mAPI calculation were obtained at the same time point, using the first measurement within 12 hours of CS diagnosis. Measurements were excluded if a tMCS device was in place at or before the time of measurement.

Patients with AMI‐CS meeting the eligibility criteria were assigned to the API or mAPI cohort depending on available hemodynamic data. Indexes were evaluated both as continuous variables and as binary variables, categorizing patients into groups with values above or below their respective median values (Figure 1).

**Figure 1:**
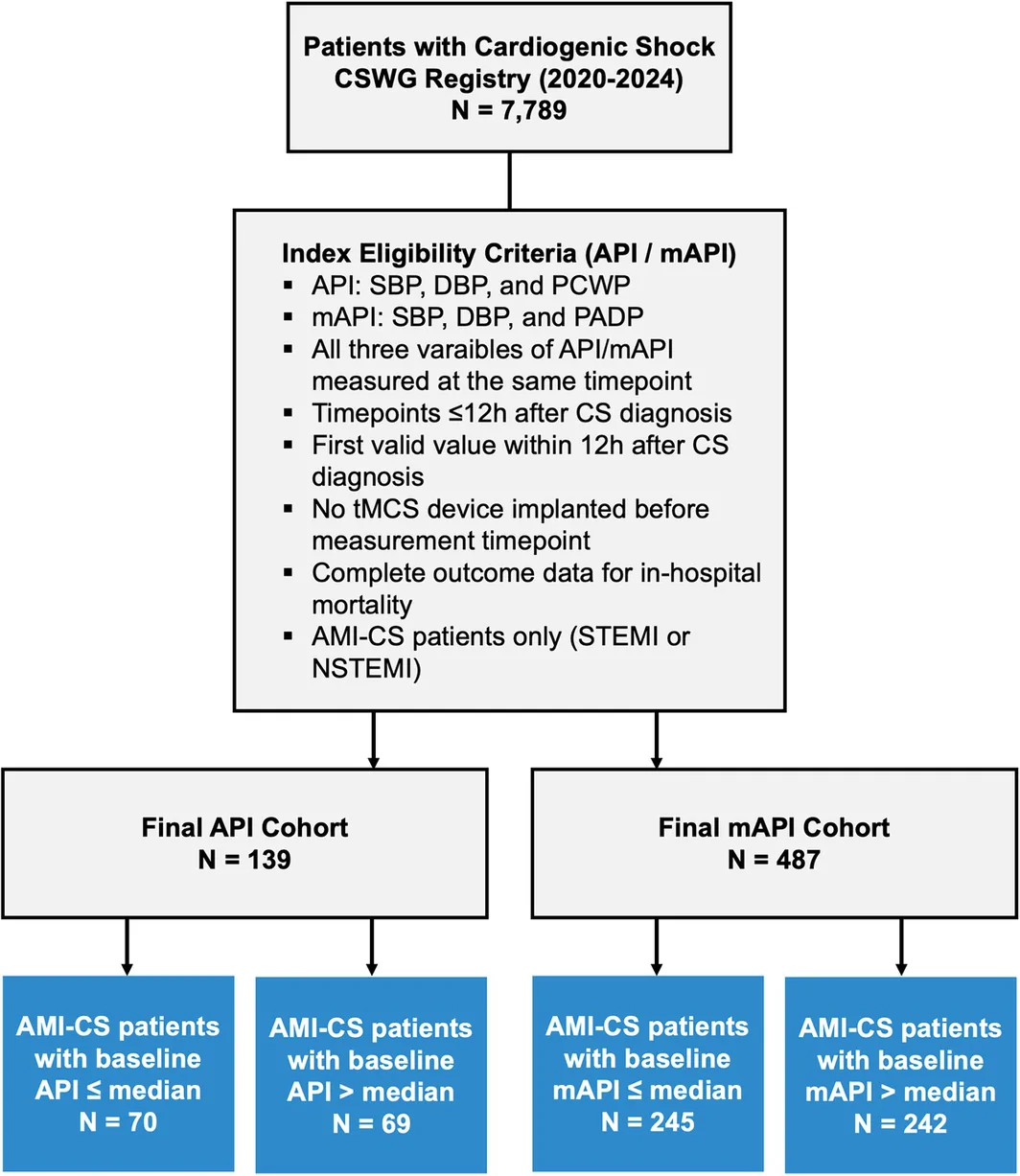
Study flowchart of patient selection for API and mAPI analysis in AMI‐CS. AMI‐CS indicates acute myocardial infarction‐related cardiogenic shock; API, arterial pulsatility index; CS, cardiogenic shock; CSWG, Cardiogenic Shock Working Group; DBP, diastolic blood pressure; mAPI, modified arterial pulsatility index; NSTEMI, non–ST‐segment–elevation myocardial infarction; PADP, pulmonary artery diastolic pressure; PCWP, pulmonary capillary wedge pressure; SBP, systolic blood pressure; STEMI, ST‐segment–elevation myocardial infarction; and tMCS, temporary mechanical circulatory support.

### Outcome

The primary outcome was in‐hospital mortality. For all analyses, only patients with complete data for this outcome were analyzed. Secondary outcomes included in‐hospital heart replacement therapy, defined as implantation of a durable left ventricular assist device (LVAD) or heart transplantation during the index hospitalization. Native heart survival was defined as discharge from the index hospitalization without heart replacement therapy. Major in‐hospital complications were captured, including stroke, in‐hospital cardiac arrest, limb ischemia, acute kidney injury, bleeding requiring surgery or transfusion, and hemolysis.

### Statistical Analysis

Binary variables are presented as absolute numbers and relative frequencies, and comparisons were conducted using Pearson χ^2^ test or Fisher exact test, as appropriate. Continuous variables are shown as the median with interquartile range (IQR) and analyzed using the Wilcoxon rank‐sum (Mann‐Whitney *U*) test.

To assess the correlation between API and mAPI in the AMI‐CS cohort, both Pearson correlation coefficients (for linear relationships) and Spearman correlation coefficients (for rank‐based relationships) were used. To evaluate associations between baseline clinical characteristics (independent variables) and low API or mAPI status (dependent variable, defined as ≤ versus > median), univariable logistic regression models were fitted. In addition, the relationship between left ventricular ejection fraction (LVEF) and API or mAPI was assessed using generalized additive models with cubic regression splines.

The associations between API and mAPI and in‐hospital mortality were assessed using different approaches. Univariable and multivariable logistic regression analyses were used to assess the association between index values and in‐hospital mortality. Indices were analyzed both dichotomized at the cohort median (≤ versus > median), predefined cutoffs (≤1.0, ≤1.5, ≤2.0, and ≤3.0), and as continuous predictors, scaled to represent the effect per 0.5‐unit decrease. Multivariable models were adjusted for key baseline variables, including age, sex, lactate, and creatinine. In addition, linear regression models were used to estimate the absolute change in mortality risk per 0.5‐unit decrease. Finally, API and mAPI were modeled as continuous variables using restricted cubic splines in logistic regression to visualize potential nonlinear associations with in‐hospital mortality. To visualize the predictive value for in‐hospital mortality, spline models were used to derive predicted probabilities across the full API and mAPI range, with 95% CIs back‐transformed from the logit scale.

The discriminatory performance of API and mAPI for in‐hospital mortality were quantified using the area under the receiver operating characteristic curve, both alone and in combination with readily available baseline predictors (creatinine, alanine aminotransferase, aspartate aminotransferase, lactate, age, or LVEF).

To evaluate associations between low (≤ median) versus high (> median) API or mAPI and heart replacement or advanced in‐hospital therapies, univariable logistic regression was performed for LVAD implantation, heart transplantation, tMCS escalation (0 versus ≥1 device; 1 versus ≥2 devices), mechanical ventilation, and renal replacement therapy.

Odds ratios (ORs) and 95% CIs are presented, and a *P* value <0.05 was considered statistically significant. Analyses were performed using R statistical software (version 4.4.1).

## RESULTS

### Study Cohort

Among 7789 patients enrolled in the CSWG registry between 2020 and 2024, 139 patients with AMI‐CS from 25 centers met the inclusion criteria for early API assessment, and 487 patients from 30 centers for early mAPI assessment (Figure 1).

Clinical characteristics of the overall cohorts and stratified by median API (2.1; IQR, 1.3–3.2) and mAPI (1.7; IQR, 1.0–2.8) are presented in Tables 1 and 2. Median age was 63 (IQR, 58–70) years in the API cohort and 64 (IQR, 55.0–71.0) years in the mAPI cohort; 31 (22.3%) and 127 (26.1%) were women, respectively. ST‐segment–elevation myocardial infarction was present in 88 (63.3%) and 320 (65.7%) patients, respectively, whereas the remaining patients were classified as non–ST‐segment–elevation myocardial infarction. Median lactate was 1.8 (IQR, 1.2–3.4) mmol/L and 2.0 (IQR, 1.2–4.1) mmol/L, creatinine 1.5 (IQR, 1.0–2.4) mg/dL and 1.4 (IQR, 1.0–2.2) mg/dL, and baseline LVEF 22.5% (IQR, 17.5%–35%) and 22.5% (IQR, 17%–35%) in the API and mAPI cohorts, respectively. Prior out‐of‐hospital cardiac arrest occurred in 21.9% in the API and 24.3% in the mAPI cohort.

**Table 1:** Characteristics of Patients With Acute Myocardial Infarction‐Cardiogenic Shock Stratified by Median API

Characteristic	Overall	API ≤2.1	API >2.1
	N=139	N=70	N=69
Demographics			
Sex			
Women	31/139 (22.3%)	9/70 (12.9%)	22/69 (31.9%)
Men	108/139 (77.7%)	61/70 (87.1%)	47/69 (68.1%)
Age, y	63.0 (58.0–70.0)	63.5 (58.0–70.0)	63.0 (57.0–71.0)
BMI, kg/m^2^	28.6 (24.9–32.5)	29.1 (25.2–33.2)	28.2 (23.9–31.4)
Medical history			
Hypertension	93/139 (66.9%)	46/70 (65.7%)	47/69 (68.1%)
Diabetes	74/139 (53.2%)	39/70 (55.7%)	35/69 (50.7%)
Atrial fibrillation/flutter	16/139 (11.5%)	12/70 (17.1%)	4/69 (5.8%)
Chronic kidney disease	26/139 (18.7%)	17/70 (24.3%)	9/69 (13.0%)
Peripheral vascular disease	14/138 (10.1%)	10/70 (14.3%)	4/68 (5.9%)
COPD	8/138 (5.8%)	6/70 (8.6%)	2/68 (2.9%)
Asthma	9/132 (6.8%)	3/68 (4.4%)	6/64 (9.4%)
Anemia	15/131 (11.5%)	6/66 (9.1%)	9/65 (13.8%)
History of stroke/TIA	13/139 (9.4%)	7/70 (10.0%)	6/69 (8.7%)
Prior CAD	54/127 (42.5%)	33/66 (50.0%)	21/61 (34.4%)
History of HF	43/138 (31.2%)	24/69 (34.8%)	19/69 (27.5%)
History of MI	36/139 (25.9%)	21/70 (30.0%)	15/69 (21.7%)
Laboratories			
Lactate, mEq/L	1.8 (1.2–3.4)	1.8 (1.1–3.5)	1.8 (1.3–3.3)
ALT, IU/L	88.5 (38.0–273.0)	91.0 (43.0–276.0)	77.0 (33.0–241.0)
AST, IU/L	189.0 (67.0–490.0)	231.0 (58.0–594.0)	180.0 (92.0–413.0)
Serum creatinine, mg/dL	1.5 (1.0–2.4)	1.6 (1.0–2.4)	1.5 (1.0–2.2)
Echocardiography			
LVEF baseline	22.5 (17.5–35.0)	22.0 (17.5–32.5)	27.5 (17.5–40.0)
Vitals			
SBP	107 (97–125)	105 (96–114)	115 (102–132)
DBP	68 (57–82)	72 (60–86)	64 (53–78)
Heart rate, bpm	95 (81–105)	95 (82–106)	96 (77–103)
MAP	81 (72–95)	81 (72–94)	83 (72–97)
API	2.1 (1.3–3.2)	1.3 (0.8–1.6)	3.2 (2.6–4.4)
OHCA/SCAI shock stage/transferred			
OHCA	30/137 (21.9%)	14/70 (20.0%)	16/67 (23.9%)
SCAI B baseline	4/108 (3.7%)	2/57 (3.5%)	2/51 (3.9%)
SCAI C baseline	35/108 (32.4%)	15/57 (26.3%)	20/51 (39.2%)
SCAI D baseline	34/108 (31.5%)	21/57 (36.8%)	13/51 (25.5%)
SCAI E baseline	35/108 (32.4%)	19/57 (33.3%)	16/51 (31.4%)
Treatments during hospital course			
No. of maximum drugs during stay			
1	12/69 (17.4%)	9/38 (23.7%)	3/31 (9.7%)
2	17/69 (24.6%)	8/38 (21.1%)	9/31 (29.0%)
3	21/69 (30.4%)	10/38 (26.3%)	11/31 (35.5%)
≥4	14/69 (20.3%)	8/38 (21.1%)	6/31 (19.4%)
VA‐ECMO	27/139 (19.4%)	17/70 (24.3%)	10/69 (14.5%)
Impella CP	6/139 (11.5%)	11/70 (15.7%)	5/69 (7.2%)
Impella 5.5	25/139 (18.0%)	14/70 (20.0%)	11/69 (15.9%)
IABP	17/139 (12.2%)	9/70 (12.9%)	8/69 (11.6%)
Mechanical ventilation	106/139 (76.3%)	58/70 (82.9%)	48/69 (69.6%)
RRT	36/139 (25.9%)	20/70 (28.6%)	16/69 (23.2%)

Binary variables are presented as absolute numbers and relative frequencies. Continuous variables are shown as median with interquartile range. ALT indicates alanine aminotransferase; API, arterial pulsatility index; AST, aspartate aminotransferase; BMI, body mass index; CAD, coronary artery disease; COPD, chronic obstructive pulmonary disease; DBP, diastolic blood pressure; HF, heart failure; IABP, intra‐aortic balloon pump; LVEF, left ventricular ejection fraction; MAP, mean arterial pressure; MI, myocardial infarction; OHCA, out‐of‐hospital cardiac arrest; RRT, renal replacement therapy; SBP, systolic blood pressure; SCAI, Society for Cardiovascular Angiography and Interventions; TIA, transient ischemic attack; and VA‐ECMO, veno‐arterial extracorporeal membrane oxygenation.

**Table 2:** Characteristics of Patients With Acute Myocardial Infarction‐Cardiogenic Shock Stratified by Median mAPI

Characteristic	Overall	mAPI ≤1.7	mAPI >1.7
	N=487	N=245	N=242
Demographics			
Sex			
Women	127/487 (26.1%)	54/245 (22.0%)	73/242 (30.2%)
Men	360/487 (73.9%)	191/245 (78.0%)	169/242 (69.8%)
Age, y	64.0 (55.0–71.0)	60.0 (52.0–69.0)	67.0 (59.0–73.0)
BMI, kg/m^2^	28.4 (24.9–32.5)	28.6 (24.9–32.8)	28.3 (25.0–32.3)
Medical history			
Hypertension	318/485 (65.6%)	148/245 (60.4%)	170/240 (70.8%)
Diabetes	224/485 (46.2%)	108/245 (44.1%)	116/240 (48.3%)
Atrial fibrillation/flutter	51/485 (10.5%)	28/245 (11.4%)	23/240 (9.6%)
Chronic kidney disease	77/485 (15.9%)	32/245 (13.1%)	45/240 (18.8%)
Peripheral vascular disease	45/484 (9.3%)	20/244 (8.2%)	25/240 (10.4%)
COPD	37/484 (7.6%)	21/244 (8.6%)	16/240 (6.7%)
Asthma	25/460 (5.4%)	8/230 (3.5%)	17/230 (7.4%)
Anemia	52/464 (11.2%)	21/229 (9.2%)	31/235 (13.2%)
History of stroke/TIA	46/486 (9.5%)	23/245 (9.4%)	23/241 (9.5%)
Prior CAD	197/448 (44.0%)	94/226 (41.6%)	103/222 (46.4%)
History of HF	130/481 (27.0%)	59/242 (24.4%)	71/239 (29.7%)
History of MI	112/483 (23.2%)	53/244 (21.7%)	59/239 (24.7%)
Laboratories			
Lactate, mEq/L	2.0 (1.2–4.1)	2.2 (1.4–4.7)	1.8 (1.2–3.4)
ALT, IU/L	82.0 (41.0–219.0)	92.0 (45.0–244.0)	72.0 (35.0–178.0)
AST, IU/L	210.0 (75.0–529.0)	248.0 (90.0–620.0)	185.0 (62.0–409.0)
Serum creatinine, mg/dL	1.4 (1.0–2.2)	1.5 (1.0–2.4)	1.4 (1.1–2.1)
Echocardiography			
LVEF baseline	22.5 (17.0–35.0)	20.0 (15.0–30.0)	27.0 (19.5–40.0)
Vitals			
SBP	107 (93–125)	100 (84–112)	116 (104–133)
DBP	69 (58–82)	73 (61–87)	64 (55–78)
Heart rate, bpm	92 (77–105)	95 (80–107)	89 (75–103)
MAP	82 (73–95)	82 (72–95)	82 (73–95)
mAPI	1.7 (1.0–2.8)	1.0 (0.7–1.4)	2.8 (2.2–3.8)
OHCA/SCAI shock stage/transferred			
OHCA	117/481 (24.3%)	66/243 (27.2%)	51/238 (21.4%)
SCAI B baseline	26/389 (6.7%)	8/198 (4.0%)	18/191 (9.4%)
SCAI C baseline	118/389 (30.3%)	46/198 (23.2%)	72/191 (37.7%)
SCAI D baseline	110/389 (28.3%)	68/198 (34.3%)	42/191 (22.0%)
SCAI E baseline	135/389 (34.7%)	76/198 (38.4%)	59/191 (30.9%)
Treatments during hospital course			
No. of maximum drugs during stay			
1	46/265 (17.4%)	18/141 (12.8%)	28/124 (22.6%)
2	65/265 (24.5%)	39/141 (27.7%)	26/124 (21.0%)
3	91/265 (34.3%)	48/141 (34.0%)	43/124 (34.7%)
≥4	56/265 (21.1%)	35/141 (24.8%)	21/124 (16.9%)
VA‐ECMO	96/487 (19.7%)	60/245 (24.5%)	36/242 (14.9%)
Impella CP	40/487 (8.2%)	23/245 (9.4%)	17/242 (7.0%)
Impella 5.5	93/487 (19.1%)	58/245 (23.7%)	35/242 (14.5%)
IABP	48/487 (9.9%)	24/245 (9.8%)	24/242 (9.9%)
Mechanical ventilation	397/485 (81.9%)	207/243 (85.2%)	190/242 (78.5%)
RRT	121/487 (24.8%)	68/245 (27.8%)	53/242 (21.9%)

Binary variables are presented as absolute numbers and relative frequencies. Continuous variables are shown as median with interquartile range. ALT indicates alanine aminotransferase; AST, aspartate aminotransferase; BMI, body mass index; CAD, coronary artery disease; COPD, chronic obstructive pulmonary disease; DBP, diastolic blood pressure; HF, heart failure; IABP, intra‐aortic balloon pump; LVEF, left ventricular ejection fraction; MAP, mean arterial pressure; mAPI, modified arterial pulsatility index; MI, myocardial infarction; OHCA, out‐of‐hospital cardiac arrest; RRT, renal replacement therapy; SBP, systolic blood pressure; SCAI, Society for Cardiovascular Angiography and Interventions; TIA, transient ischemic attack; and VA‐ECMO, veno‐arterial extracorporeal membrane oxygenation.

API was significantly correlated with mAPI (Pearson *r*=0.75, *P*<0.001; Spearman ρ=0.78, *P*<0.001; Figure S1). PADP and mean pulmonary artery pressure were moderately correlated with PCWP (Figure S2).

### Association Among API, mAPI, and Clinical Characteristics

In the API cohort, lower API (≤ median) was significantly associated with male sex (OR, 3.17 [95% CI, 1.37–7.86]; *P*=0.009). Other characteristics, including comorbidity burden, markers of hypoperfusion, and shock severity, did not differ significantly among groups in the context of the limited sample size of this cohort.

Lower mAPI (≤ median) was associated with younger age (OR, 0.42 [95% CI, 0.29–0.61]; *P*<0.001), male sex (OR, 1.53 [95% CI, 1.02–2.31]; *P*=0.042), lower baseline LVEF (OR, 2.47 [95% CI, 1.63–3.74]; *P*<0.001), and higher baseline lactate levels (OR, 1.56 [95% CI, 1.07–2.73]; *P*=0.020). Likewise, lower mAPI was associated with higher transaminases (alanine aminotransferase: OR, 1.59 [95% CI, 1.09–2.32]; *P*=0.015; aspartate aminotransferase: OR, 1.55 [95% CI, 1.06–2.26]; *P*=0.023).

The overall distributions of API and mAPI across baseline SCAI stages showed no major differences (Figure S3A and S3B). A lower mAPI was associated with lower odds of presenting in lower SCAI stages B (OR, 0.42 [95% CI, 0.20–0.96]; *P*=0.046) and C (OR, 0.55 [95% CI, 0.36–0.85]; *P*=0.005), and was associated with higher SCAI stages (SCAI D: OR, 1.83 [95% CI, 1.19–2.84]; *P*=0.006; E: OR, 1.40 [95% CI, 0.99–2.08]; *P*=0.102; Figure 2).

**Figure 2:**
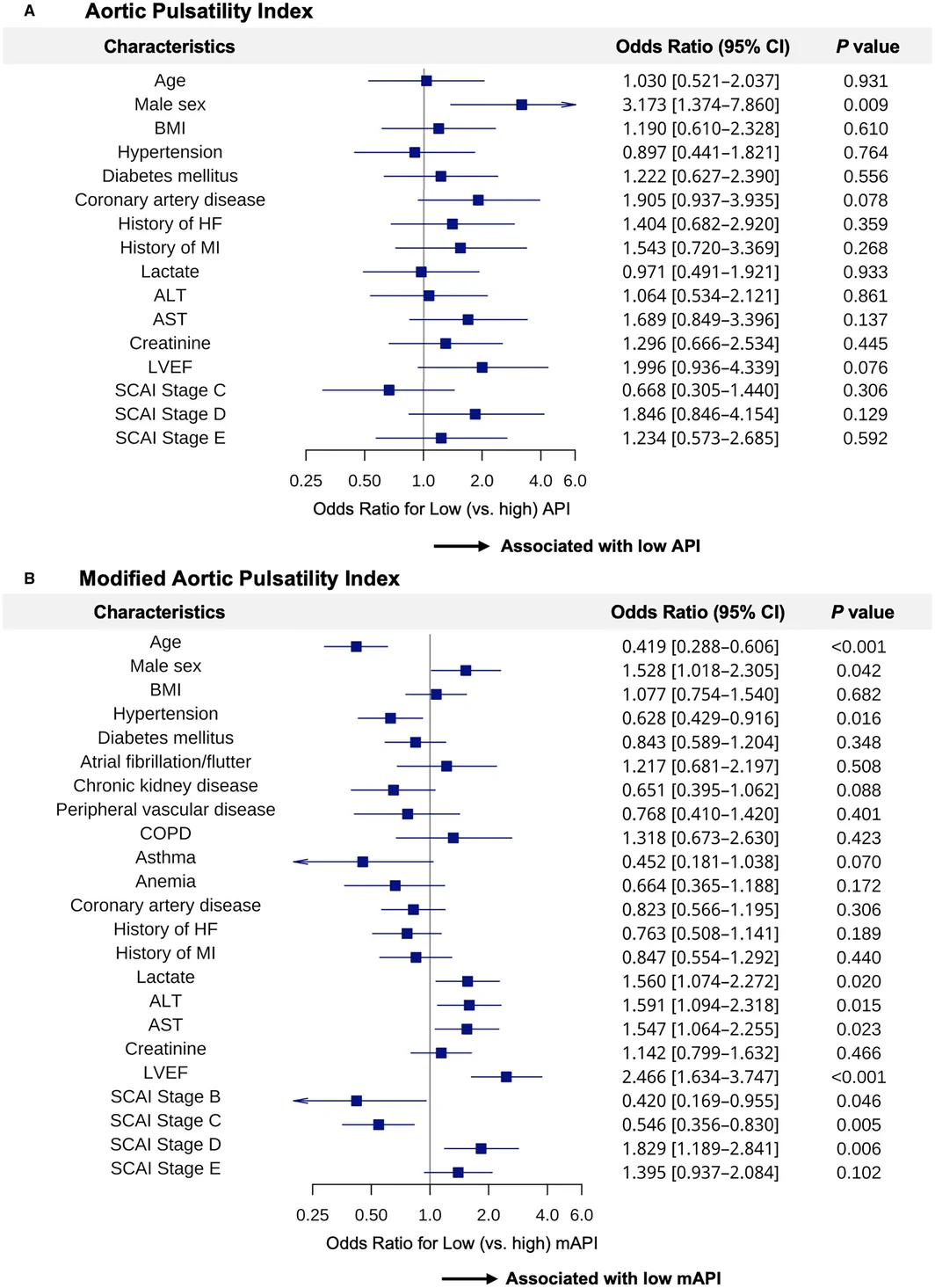
Baseline characteristics associated with low API and mAPI in AMI‐CS. Univariable logistic regression models were used to assess associations between clinical characteristics and a low API (**A**) or mAPI (**B**), defined as ≤ vs > median. ALT indicates alanine aminotransferase; AMI‐CS, acute myocardial infarction‐related cardiogenic shock; API, arterial pulsatility index; AST, aspartate aminotransferase; BMI, body mass index; COPD, chronic obstructive pulmonary disease; HF, heart failure; LVEF, left ventricular ejection fraction; mAPI, modified arterial pulsatility index; MI, myocardial infarction; and SCAI, Society for Cardiovascular Angiography and Interventions.

In spline analyses, API increased with rising LVEF but plateaued at values above ≈2.5, with steeper slopes observed at the lower range of each index. In contrast, mAPI demonstrated an approximately linear correlation with LVEF (Figure S4A and S4B).

### Associations Among API, mAPI, and Outcomes

Crude in‐hospital mortality was significantly higher in patients with lower API values (≤ median) compared with those above (47.1% versus 24.6%, *P*=0.010), and likewise among patients with lower mAPI (44.1% versus 32.2%, *P*=0.009; Figure 3A and 3B). Crude in‐hospital mortality progressively increased with higher SCAI stage, with consistently higher but not statistically significant rates in patients with lower compared with higher API and mAPI values (Figure S5A and S5B). Native heart survival was more frequently observed among patients with higher API and mAPI (59.4% versus 41.4%, *P*=0.051; and 59.1% versus 41.2%, *P*<0.001, respectively; Figure 3A and 3B), compared with lower API and mAPI values.

**Figure 3:**
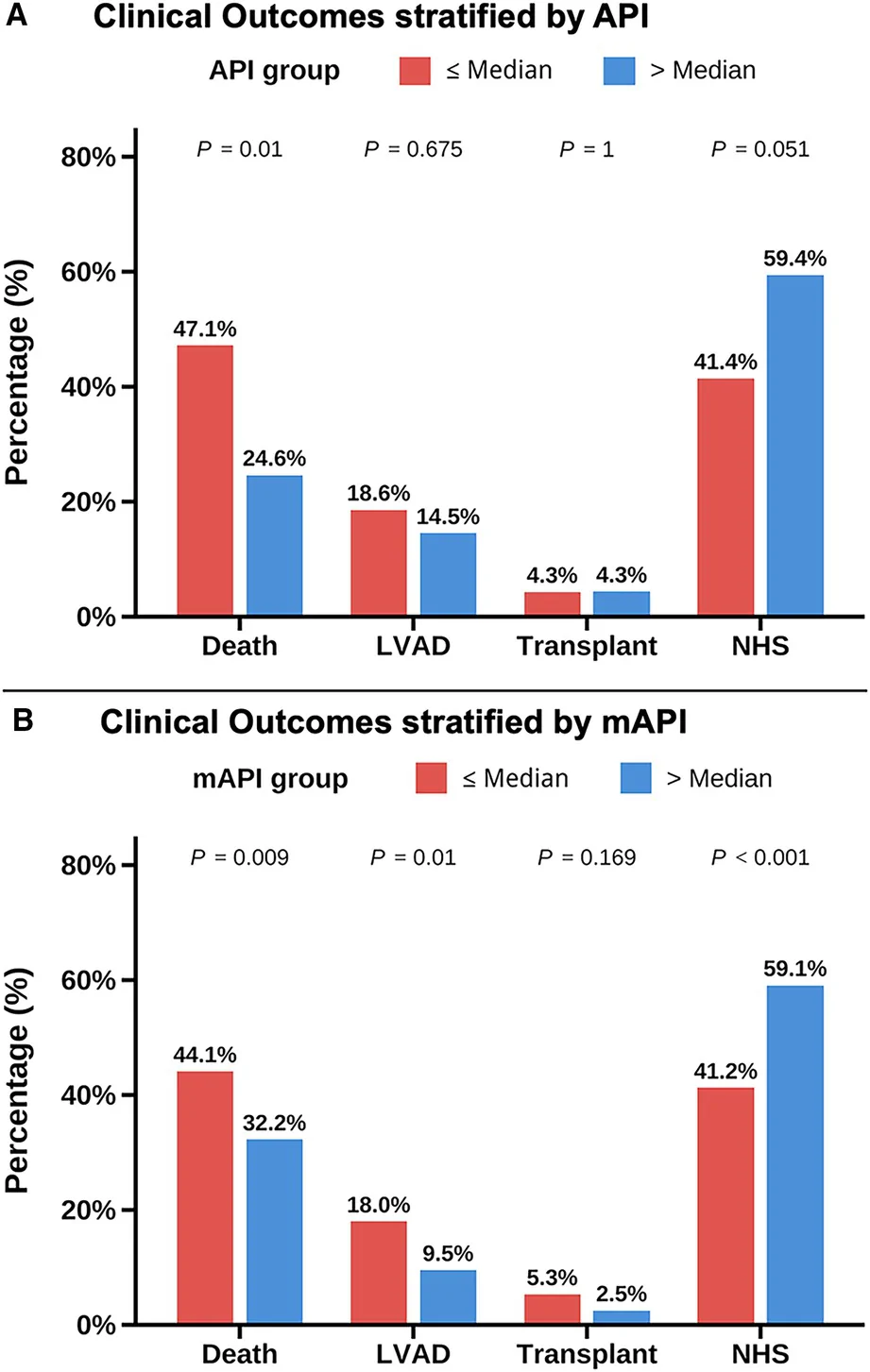
Crude clinical outcomes in patients with AMI‐CS stratified by median API and mAPI. **A**, Crude in‐hospital outcomes stratified by median API. **B**, Crude in‐hospital outcomes stratified by median mAPI. Outcomes included in‐hospital mortality (death), durable LVAD implantation, heart transplantation, and NHS. *P* values were calculated using χ^2^ tests. API indicates arterial pulsatility index; LVAD, left ventricular assist device; mAPI, modified arterial pulsatility index; and NHS, native heart survival.

In AMI‐CS, lower API was independently associated with 173% higher relative odds for in‐hospital mortality (OR, 2.728 [95% CI, 1.341–5.705]; *P*=0.006); lower mAPI was associated with 66% higher relative odds for in‐hospital mortality (OR, 1.657 [95% CI, 1.147–2.402]; *P*=0.007). Each 0.5 unit decrease in API and mAPI was associated with higher odds of in‐hospital mortality (API: OR, 1.133 [95% CI, 1.017–1.286]; *P*=0.036; mAPI: OR, 1.069 [95% CI, 1.011–1.137]; *P*=0.024), consistent with linear regression analyses (API: β=0.024 [95% CI, 0.002–0.045]; *P*=0.030; mAPI: β=0.014 [95% CI, 0.002–0.026]; *P*=0.022). After adjustment for age, sex, lactate, and creatinine, associations for both API and mAPI were attenuated but remained directionally consistent, indicating a clear trend toward higher in‐hospital mortality with lower index values (Figure 4A and 4B). Exploratory threshold analyses demonstrated higher in‐hospital mortality at lower API and mAPI values, with the strongest and most consistent associations observed below approximately 1.5 to 2.0 (Table 3).

**Figure 4:**
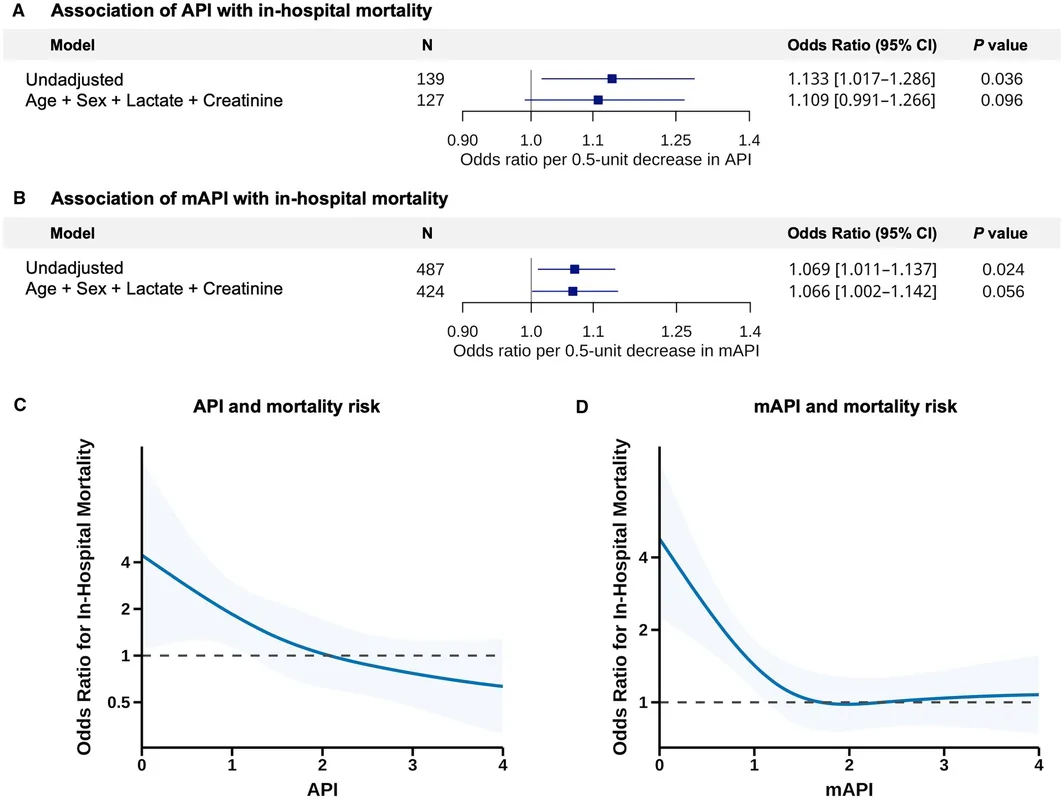
Association between API/mAPI and in‐hospital mortality. **A**, Multivariable logistic regression models were used to assess the association between API (per 0.5‐unit decrease) and in‐hospital mortality, adjusted for age, sex, baseline lactate, and baseline creatinine. **B**, Corresponding associations for the mAPI. **C**, Restricted cubic spline illustrating the continuous association between API and the odds of in‐hospital mortality. **D**, Corresponding spline analysis for mAPI. Blue lines represent estimated odds ratios; shaded areas represent 95% CIs. API indicates arterial pulsatility index; and mAPI, modified arterial pulsatility index.

**Table 3:** Associations of API and mAPI With In‐Hospital Mortality Across Predefined Cutoffs

Variable	Cutoff	Model	N	OR	95% CI	*P* value
API	≤1	Unadjusted	139	2.026	(0.827–4.979)	0.120
API	≤1	Adjusted	127	1.696	(0.661–4.339)	0.267
API	≤1.5	Unadjusted	139	2.307	(1.117–4.807)	0.024
API	≤1.5	Adjusted	127	2.121	(0.963–4.738)	0.063
API	≤2	Unadjusted	139	2.876	(1.416–5.990)	0.004
API	≤2	Adjusted	127	3.065	(1.388–7.056)	0.007
API	≤3	Unadjusted	139	1.933	(0.871–4.572)	0.116
API	≤3	Adjusted	127	1.621	(0.677–4.087)	0.288
mAPI	≤1	Unadjusted	487	1.967	(1.300–2.982)	0.001
mAPI	≤1	Adjusted	424	1.856	(1.155–2.990)	0.011
mAPI	≤1.5	Unadjusted	487	1.813	(1.253–2.630)	0.002
mAPI	≤1.5	Adjusted	424	1.889	(1.237–2.897)	0.003
mAPI	≤2	Unadjusted	487	1.560	(1.073–2.279)	0.021
mAPI	≤2	Adjusted	424	1.652	(1.078–2.551)	0.022
mAPI	≤3	Unadjusted	487	1.165	(0.739–1.859)	0.515
mAPI	≤3	Adjusted	424	1.261	(0.761–2.117)	0.372

Unadjusted and adjusted associations among API, mAPI, and in‐hospital mortality across predefined index ranges. Multivariable analyses were adjusted for age, sex, baseline lactate, and baseline creatinine. API indicates arterial pulsatility index; mAPI, modified arterial pulsatility index; and OR, odds ratio.

Restricted cubic spline analyses demonstrated a graded, inverse association between both API and mAPI and in‐hospital mortality (OR and predicted absolute risks), with the strongest effects observed at values <2 (Figure 4C and 4D; Figure S6A and S6B). For mAPI, the decline in predicted mortality risk attenuated at higher values, resulting in a plateau beyond ≈2, whereas for API a more continuous inverse association was observed.

In discriminative performance analysis for in‐hospital mortality in the AMI‐CS cohort, the unadjusted area under the receiver operating characteristic curve was 0.622 (95% CI, 0.512–0.732) for API, and 0.581 (95% CI, 0.527–0.634) for mAPI. API and mAPI demonstrated discrimination comparable with systolic blood pressure (Figure S7A and S7B). The discriminative performance of both indices improved when combined with clinically readily available individual baseline predictors (creatinine, alanine aminotransferase, aspartate aminotransferase, lactate, age, or LVEF; Figure 5A and 5B). Particularly, combinations with creatinine and lactate showed an increased discriminative performance for both indices (API+creatinine: area under the receiver operating characteristic curve, 0.669; [95% CI, 0.575–0.764]; API+lactate: 0.647 [95% CI, 0.549–0.745]; mAPI + creatinine: 0.648 [95% CI, 0.597–0.698]; mAPI + lactate: 0.674 [95% CI, 0.623–0.726]), with analyses performed both in unadjusted, median‐stratified models.

**Figure 5:**
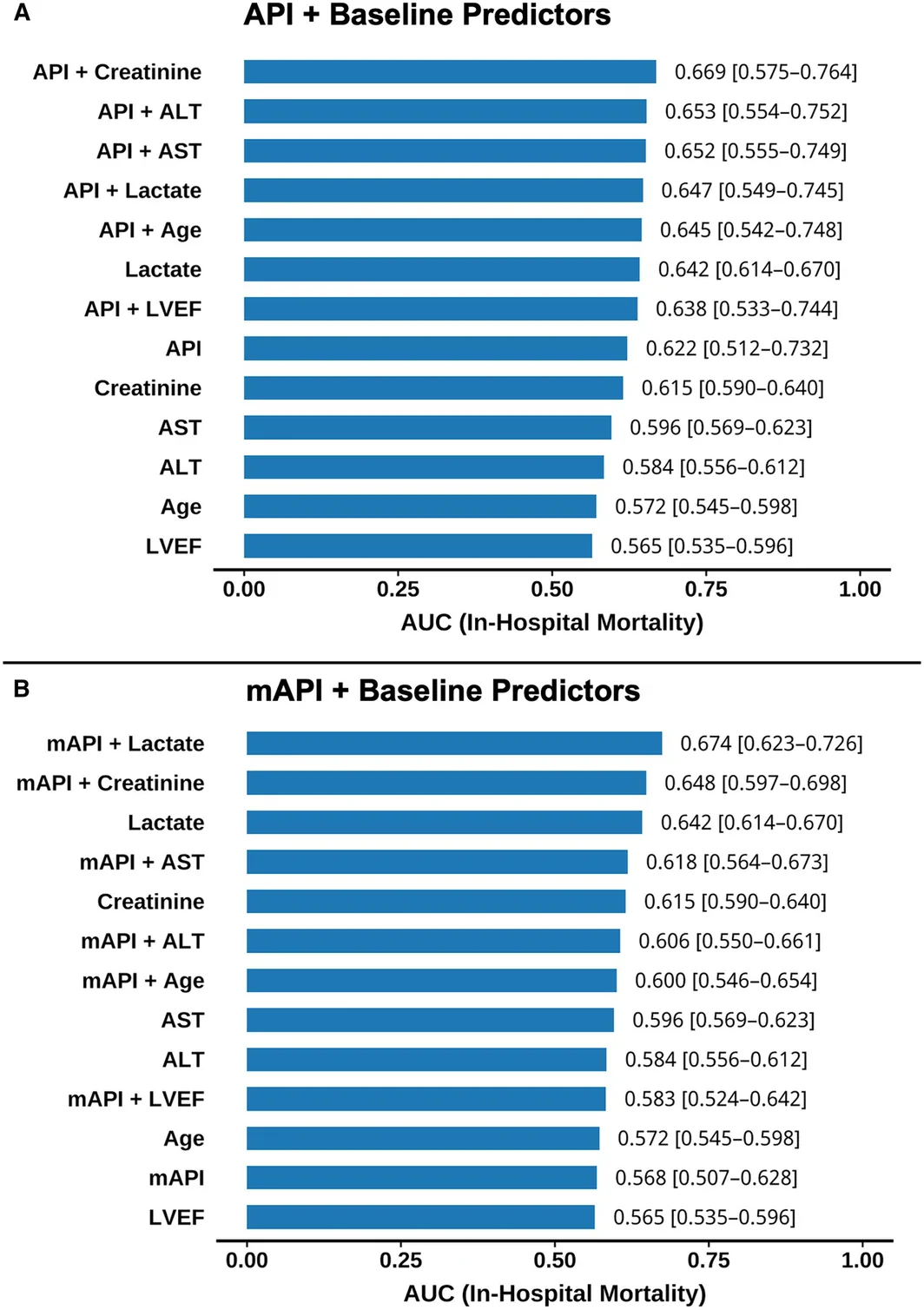
Discriminative performance of API and mAPI in combination with baseline predictors for in‐hospital mortality in AMI‐CS. **A**, AUCs for API alone and in combination with clinically readily available individual baseline predictors (creatinine, AST, lactate, age, or LVEF) to predict in‐hospital mortality. **B**, Corresponding AUCs for mAPI. Bars represent AUC values with 95% CIs. ALT indicates alanine aminotransferase; AMI‐CS, acute myocardial infarction‐related cardiogenic shock; API, arterial pulsatility index; AST, aspartate aminotransferase; AUC, area under the receiver operating characteristic curve; LVEF, left ventricular ejection fraction; and mAPI, modified arterial pulsatility index.

### Associations Among API, mAPI, and In‐Hospital Treatments

The association among low API, low mAPI, and in‐hospital treatments in AMI‐CS is illustrated in Figure 6A and 6B. Although there were no significant associations between low API and the in‐hospital use of treatments, a lower mAPI was independently associated with higher odds for durable LVAD implantation (OR, 2.084 [95% CI, 1.227–3.626]; *P*=0.008) and escalation with tMCS (OR, 1.859 [95% CI, 1.295–2.679]; *P*<0.001). Trends were also observed toward higher odds for use of ≥2 mechanical circulatory support devices and of mechanical ventilation.

**Figure 6:**
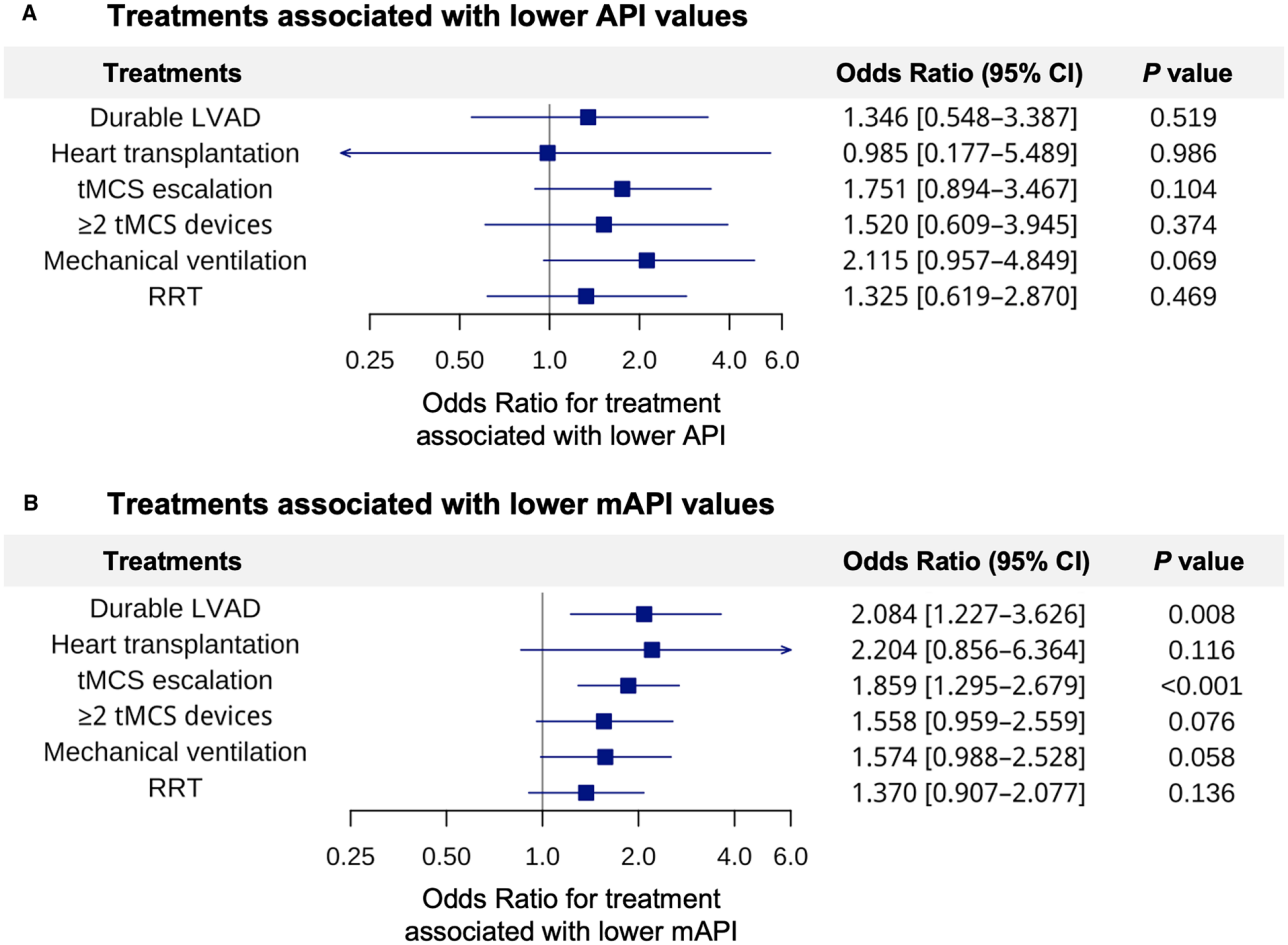
Association between API/mAPI and treatments in AMI‐CS. **A**, Logistic regression models were used to assess the association between the API and advanced therapies, durable LVAD implantation, heart transplantation, escalation with tMCS, use of ≥2 tMCS devices, mechanical ventilation, and renal replacement therapy during index hospitalization. **B**, Corresponding associations for the mAPI. AMI‐CS indicates acute myocardial infarction‐related cardiogenic shock; API, arterial pulsatility index; LVAD, left ventricular assist device; mAPI, modified arterial pulsatility index; RRT, renal replacement therapy; and tMCS, temporary mechanical circulatory support.

## DISCUSSION

In this large, multicenter, contemporary cohort of patients with AMI‐CS, lower API and mAPI values were associated with higher in‐hospital mortality, with the strongest risk gradient observed at values <≈2. Indexes were linked to elevated markers of hypoperfusion, higher SCAI shock stages, the need for escalation to tMCS, and durable LVAD implantation. Although API and mAPI were strongly correlated, direct comparisons between the 2 indices are limited by the smaller sample size of the API cohort. The newly applied mAPI may provide complementary prognostic information by relying on a more readily obtainable invasive hemodynamic measure in clinical settings compared with API. To our knowledge, these data provide the first comprehensive characterization of API and mAPI in a large, real‐world, contemporary multicenter AMI‐CS cohort, suggesting that these indices may contribute to early risk assessment.

### Associations of API and mAPI With Clinical Characteristics

Early recognition and triage of patients with CS increasingly rely on structured algorithms incorporating shock severity staging, phenotyping, risk modifiers, and hemodynamic profiling to guide clinical management.^2^, ^12^, ^21^, ^22^, ^23^ AMI‐CS typically presents abruptly with hypotension and early hypoperfusion, driving a rapid clinical trajectory, reflecting acutely impaired LV contractility and elevated filling pressures.^12^, ^24^ LV end‐diastolic pressure is a well‐established index of risk for development of in‐hospital heart failure and both short‐ and long‐term mortality in AMI.^25^ Similar to the pulmonary artery pulsatility index for assessing right heart failure, the API, as a novel measurement in CS, integrates LV contractile performance with filling pressures by relating systemic pulse pressure to PCWP.^17^, ^19^, ^26^ Systemic pulse pressure can correlate with stroke volume under the assumption of relatively constant arterial compliance. PCWP, reflecting LV end‐diastolic pressure, also serves as an index of preload. Whether early API carries prognostic value in AMI‐CS has not been examined in clinical settings and was therefore further investigated in this study. In addition, an mAPI is introduced here, for the first time, as a pragmatic alternative to broaden applicability.

In this large, multicenter study, API was strongly correlated with mAPI. Although both indices capture the interplay of pulsatility and filling pressures, mAPI substitutes PCWP with PADP. Our data suggest that mAPI may serve as a valid surrogate when PCWP measurements are unavailable, particularly in the acute setting where wedge acquisition is technically challenging. Importantly, both indices can be derived without cardiac output measurement, thereby avoiding potential error from thermodilution or Fick‐based methods and providing a simplified approach to hemodynamic assessment in acute care.

API showed only numerical trends toward elevated markers of hypoperfusion and higher shock severity, likely reflecting the limited cohort size. Lower mAPI values were consistently associated with decreased LVEF, higher lactate and transaminase levels, and advanced SCAI stages D and E. Lower LVEF reflects diminished contractile performance, which subsequently lowers systemic pulse pressure while increasing left‐sided filling pressures, changes that directly translate into lower API values. Our findings further suggest that baseline mAPI may capture systemic hypoperfusion and impending progression to refractory shock. As novel indexes, API and mAPI may therefore facilitate earlier recognition of hypoperfusion before overt clinical signs are present. Our results emphasize the need for further research into deeper hemodynamic phenotyping and correlation with markers of shock severity, as well as for defining clinically relevant hypoperfusion thresholds to support therapeutic decision‐making.

### Associations of API and mAPI With Clinical Outcomes

In patients with CS, PAC use allows early identification of significant hemodynamic compromise, facilitates differentiation among isolated left‐, right‐, or biventricular failure, and helps optimize volume status, vasoactive therapy, timely tMCS escalation, and weaning.^12^, ^13^, ^15^, ^27^ PAC use in CS has been shown to be associated with lower mortality.^13^, ^16^, ^28^ In the SHOCK (Should We Emergently Revascularize Occluded Coronaries for Cardiogenic Shock) trial, cardiac power (cardiac output × mean arterial pressure) was strongly associated with in‐hospital mortality.^29^ Complete hemodynamic assessment obtained before tMCS initiation has been linked to improved survival, particularly in patients with advanced SCAI stages.^13^ However, prior CS studies on hemodynamics have primarily focused on mean arterial pressure, right atrial pressure, PCWP, cardiac index, cardiac power output, and the pulmonary artery pulsatility index.^13^, ^16^, ^27^, ^28^, ^30^ The prognostic relevance of API has not been examined in these studies.

In this study, lower API and mAPI values were associated with higher in‐hospital mortality among patients with AMI‐related cardiogenic shock, demonstrating an inverse relationship between APIs and mortality risk. This relationship was consistent when indices were modeled as continuous variables and persisted after adjustment for relevant baseline clinical characteristics. Adjusted exploratory threshold analyses indicated higher in‐hospital mortality at API and mAPI values <≈2, suggesting a clinically relevant high‐risk zone rather than a discrete cut‐off.

These findings are consistent with prior studies in HF cohorts, demonstrating the predictive value of API for clinical outcomes, whereas comparable data in CS have been lacking.^17^, ^31^, ^32^ Several factors may explain our findings. First, in AMI‐CS, the extent of myocardial ischemia, particularly in transmural infarcts, limits left ventricular stroke output and consequently reduces arterial pulsatility, resulting in lower API and mAPI values. In a subanalysis of the DOREMI (Dobutamine compared with Milrinone)  trial, early increase in pulse pressure 24 hours after inotrope initiation was associated with improved outcomes in patients with SCAI stage C CS.^33^

Second, the denominator of API and mAPI reflects ventricular filling pressures. Elevated PCWP or PADP in the setting of impaired contractility depresses API and mAPI.^19^ In a recent CCCTN (Critical Care Cardiology Trials Network)  registry analysis, hemodynamic indices reflecting low cardiac output and elevated LV filling pressures alone were not associated with in‐hospital mortality in CS and the AMI‐CS subset.^19^ Ratios that integrate congestion or perfusion pressure, such as right atrial pressure/PCWP or systolic blood pressure/PCWP, appear more prognostically informative in contemporary CS cohorts.^19^

Third, compensatory peripheral vasoconstriction predominantly raises diastolic rather than systolic pressure, and thereby may fail to restore pulse pressure. Consequently, API and mAPI may remain low despite preserved mean arterial pressure, suggesting they may serve as early surrogates of impending low‐output circulatory failure.

In addition, lower mAPI was associated with a higher rate of escalation to tMCS and showed higher crude in‐hospital mortality across SCAI stages. Replication in larger data sets may clarify whether these indices delineate higher‐risk subsets within SCAI stages or signal trajectories toward worsening or recovery. Notably, the discriminative performance of both indices improved when combined with clinically readily available baseline predictors. Taken together, these considerations highlight API and mAPI as promising tools for early prognostic assessment and guiding upstream therapy.

A plateau in mortality risk was observed for mAPI at higher values. At low mAPI levels, systemic hypoperfusion appears to be the dominant driver of outcome, rendering incremental improvements in pulsatile output prognostically meaningful. Beyond a certain threshold, however, mortality may be increasingly determined by nonhemodynamic factors, including infarct size, multiorgan failure, or neurological injury, which are not directly captured by pressure‐derived indices. The larger sample size available for mAPI likely enabled detection of this nonlinear relationship. In contrast, in the smaller API cohort, the apparently more continuous inverse association may reflect limited statistical power and should therefore be interpreted with caution.

This study of API and mAPI included measurements obtained only before initiation of tMCS. During tMCS support, the interpretation and prognostic significance of these indexes is altered, because devices directly affect left ventricular filling pressures and aortic pressure pulsatility. The intra‐aortic balloon pump increases pulse pressure and would thereby increase API and mAPI, even without a true improvement in native LV function. Conversely, Impella devices unload the LV, thus decreasing PCWP and PADP, while also decreasing arterial pulse pressure; the next impact on API and mAPI are not predictable. Veno‐arterial extracorporeal membrane oxygenation generally decreases pulse pressure and has variable effects on PCWP, with values decreasing in some patients and increasing in others. Although changes of these indexes may be indicative of changes in LV performance, multiple device‐related factors (eg, pump speeds and flows) confound interpretation. Thus, during tMCS use, serial measurements to track changes of LV function might be more informative than single values. However, it must be further kept in mind that changes in device settings alter these indexes. Thus, the use of API and mAPI during tMCS use requires further investigation.

Future research should define individualized API and mAPI thresholds and evaluate these indices across CS subsets, with the goal of embedding them into decision algorithms within multidisciplinary shock team approaches.^12^, ^22^, ^34^

### Limitations

This study has several limitations. First, its nonrandomized, observational design precludes causal inference; residual confounding cannot be excluded, and the in‐hospital mortality estimates are unadjusted. Second, only short‐term, in‐hospital outcomes were available, limiting conclusions about long‐term effects. Third, hemodynamics (eg, PCWP, PADP) were obtained at clinician discretion and mostly at single time points, making selection bias and timing effects possible. In addition, blood pressure values are recorded as routine clinical measurements without consistent documentation of whether they were obtained invasively via arterial lines or noninvasively. Fourth, no prespecified or validated cutoff values for API or mAPI were applied, and findings should be interpreted across index ranges rather than discrete thresholds. Parts of the analyses included small sample sizes. In particular, the smaller API cohort may have limited statistical power to detect nonlinear relationships and stage‐dependent changes across SCAI stages, especially at higher index values.

Fewer than 10% of patients with AMI‐CS in this registry underwent complete early invasive hemodynamic assessment. Consequently, the analyzed cohort may not be fully representative of the broader AMI‐CS population, potentially limiting generalizability. At the same time, this finding underscores the limited penetration of early invasive hemodynamic profiling in contemporary real‐world AMI‐CS practice.

Finally, upstream decisions on vasoactive drugs and tMCS were made by treating physicians and likely influenced by provider preference and institutional practice patterns.

## CONCLUSIONS

In this multicenter cohort of patients with AMI‐CS, lower API and mAPI values were associated with higher in‐hospital mortality, escalation to tMCS and durable LVAD implantation, as well as with elevated markers of hypoperfusion and higher shock severity. Accordingly, API and mAPI may facilitate early risk assessment and help guide treatment in patients with AMI‐CS.

## Sources of Funding

This work was supported by institutional grants from Johnson & Johnson/Abiomed Inc., Boston Scientific Inc., Getinge Inc., LivaNova Inc. and Teleflex Inc. to Tufts Medical Center. The sponsors had no input on collection, analysis, and interpretation of the data, nor in the preparation, review, or approval of the article. Dr Sundermeyer is supported by the German Research Foundation (grant number 546376900) and received travel grants from the German Center for Cardiovascular Research.

## Disclosures

Dr Kanwar has served on the advisory boards for Abiomed Inc., Abbott Laboratories, and CorWave, and has received speaker fees from Abiomed. Dr Li has received consulting fees from Abiomed. Dr Hernandez‐Montfort has received consulting fees from Abiomed Inc. and Abbott and Boston Scientific. Dr Garan has received consulting fees from NuPulseCV and Abiomed, research support from Verantos, and received honoraria from Getinge. Dr Abraham has received consulting fees from Abbott Laboratories, Abiomed Inc., and Ancora. Dr Mahr has received consulting fees from Abbott, Abiomed, and Endotronix. Dr Burkhoff has received consulting fees from Boston Scientific, Medtronic, TherOx, and Abiomed. Dr Zweck has received travel grants and speaker honoraria from Abiomed. Dr Kapur is an employee of Johnson & Johnson MedTech. The remaining authors have no disclosures to report.

## Supporting information

Figures S1–S7
CSWG Academic Research Consortium members

STROBE checklist
