# Supplementary material for: Arterial Pulsatility Index in Acute Myocardial Infarction‐Related Cardiogenic Shock: Clinical Characteristics and Association With Outcomes
Source: J Am Heart Assoc. 2026 Aug 20;15(17):e047854. doi: 10.1161/JAHA.125.047854 (PMC13695795; doi:10.1161/JAHA.125.047854)
Supplement: Supplementary file 1 [file JAH3-15-e047854-s001.pdf]

SUPPLEMENTAL MATERIAL

Cardiogenic Shock Working Group Academic Research Consortium investigators:

Jonas Sundermeyer, MD, MHBA[1,2]; Song Li, MD[3]; Van-Khue Ton, MD, PhD[4]; Rachna Kataria, MD[5]; Elric Zweck, MD[6]; Kevin John, MD[1]; Manreet K. Kanwar, MD[7]; Jaime Hernandez-Montfort, MD, MPH[8]; Shashank S. Sinha, MD, MSc[9]; A. Reshad Garan, MD[10]; Jacob Abraham, MD[11]; Vanessa Blumer, MD[12]; Ajar Kochar, MD[13]; Karthikeyan Ranganathan, MD[14]; Gavin W. Hickey MD[15]; Mohit Pahuja, MD[16]; Scott Lundgren, MD[17]; Sandeep Nathan MD[18]; Esther Vorovich, MD[19]; Shelley Hall, MD[20]; Wissam Khalife, MD[21]; Andrew Schwartzman, MD[22]; Ju Kim, MD[23]; Oleg Alec Vishnevsky, MD[24]; Justin Fried, MD[25]; Maryjane Farr, MD[26]; Joseph Mishkin, MD[27]; I-Hui Chang, MD[28]; Onyedika Ilonze, MD[29]; Alexandra Arias, MD[30]; Jun Nakata, MD[31]; Jeffrey Marbach, MD[32]; Hiram Bezerra, MD[33]; Ann Gage, MD[34]; Joyce Wald, MD[35]; Sunu Thomas, MD[36]; Faisal Rahman, MD[37]; Amirali Masoumi, MD[38]; Aasim Afzal, MD[39]; Salman Gohar, MD[40]; Rachel Goodman, MD[1]; Karol D. Walec, BS[1]; Peter S. Natov, MD[1]; Borui Li, MA[1]; Paavni Sangal, MPH[1]; Qiuyue Kong, MS[1]; Peter Zazzali, MS, MPH[1]; Neil M. Harwani, MS[1]; Saraschandra Vallabhajosyula, MD[41]; Arvind Bhimaraj, MD[42]; M. Imran Aslam, MD[1]; Christian Said, MD[43]; Claudius Mahr, MD[3]; Daniel Burkhoff, MD, PhD[44]; and Navin K. Kapur, MD[1]

1 The Cardiovascular Center, Tufts Medical Center, Boston, Massachusetts, USA
2 Department of Cardiology, University Heart and Vascular Center Hamburg, University Medical Center Hamburg-Eppendorf, Hamburg, Germany
3 Institute for Advanced Cardiac Care, Medical City Healthcare, Dallas, Texas, USA
4 Massachusetts General Hospital, Boston, USA
5 Brown University Health Cardiovascular Institute, Providence, Rhode Island
6 Department of Cardiology, Pulmonology, and Vascular Medicine, Medical Faculty and University Hospital Düsseldorf, Heinrich-Heine-University, Düsseldorf, Germany
7 University of Chicago, Chicago, USA
8 Baylor Scott & White Health, Advanced Heart Failure Program Clinic, Temple, USA
9 Inova Heart and Vascular Institute, Inova Fairfax Campus, Falls Church, USA
10 Beth Israel Deaconess Medical Center, Boston, USA
11 Center for Cardiovascular Analytics, Research, & Data Science (CARDS), Providence St. Joseph Research Network, Portland, USA
12 Inova Heart and Vascular Institute, Inova Fairfax Campus, Falls Church, VA
13 Division of Cardiovascular Medicine, Brigham and Women's Hospital, Boston, USA
14 Cardiovascular Institute at Allegheny Health Network, Pittsburgh, USA
15 University of Pittsburgh Medical Center, Pittsburgh, USA
16 University of Oklahoma Health Science Center, Oklahoma City, USA
17 University of Nebraska Medical Center, Omaha, USA
18 University of Chicago, Chicago, USA
19 Northwestern Medicine, Chicago, USA
20 Baylor University Medical Center, Dallas, USA
21 University of Texas Medical Branch, Galveston, USA
22 Maine Medical Center, Portland, USA
23 Houston Methodist Research Institute, Houston, USA
24 Thomas Jefferson University Hospital, Philadelphia, USA
25 Columbia University Irving Medical Center, New York, USA
26 UT Southwestern, Dallas, USA
27 Atrium Health Sanger Heart and Vascular Institute, Charlotte, USA
28 Banner University Medical Center, Phoenix, USA
29 Indiana University School of Medicine, Indianapolis, USA
30 Instituto Nacional de Cardiologia Ignacio Chavez, Mexico City, Mexico
31 Nippon Medical School, Tokyo, Japan
32 Oregon Health State University, Portland, Oregon
33 Tampa General Hospital, Tampa, Florida, USA
34 TriStar Centennial Medical Center, Nashvil, USA
35 University of Pennsylvania, Philadelphia, USA
36 University of Washington Medical Center, Seattle, USA
37 Johns Hopkins University, Baltimore, USA
38 Atlantic Health System, Morristown, USA
39 Baylor Scott and White, Plano, USA
40 Baylor Scott and White, Fort Worth, USA
41 Brown University Health, Providence, USA
42 Houston Methodist Hospital, Houston, USA
43 The Victor Chang Cardiac Research Centre, Darlinghurst, New South Wales, Australia; The University of New South Wales, Kensington, New South Wales, Australia; Cardiology Department, Gosford Hospital, Central Coast Local Health District, Gosford, Australia
44 Cardiovascular Research Foundation, New York, USA

Figure S1: Correlation between API and mAPI in AMI-CS.

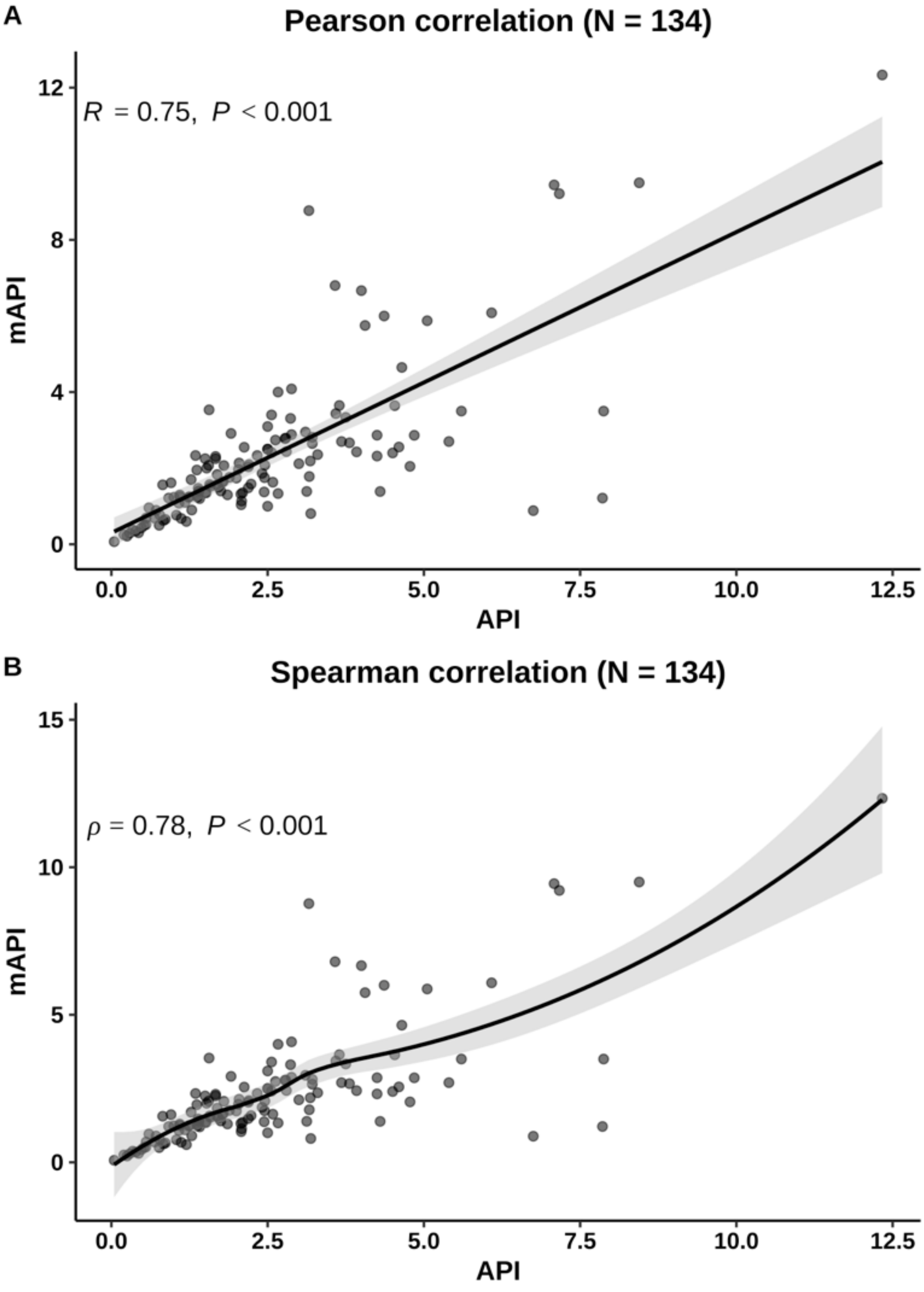

Pearson **(A)** and Spearman **(B)** correlation methods were used to assess the association between API and mAPI in patients with AMI-CS. AMI-CS = acute myocardial infarction–related cardiogenic shock; API = arterial pulsatility index; mAPI = modified arterial pulsatility index.

Figure S2: Correlation between PCWP, PADP and mPAP in AMI-CS.

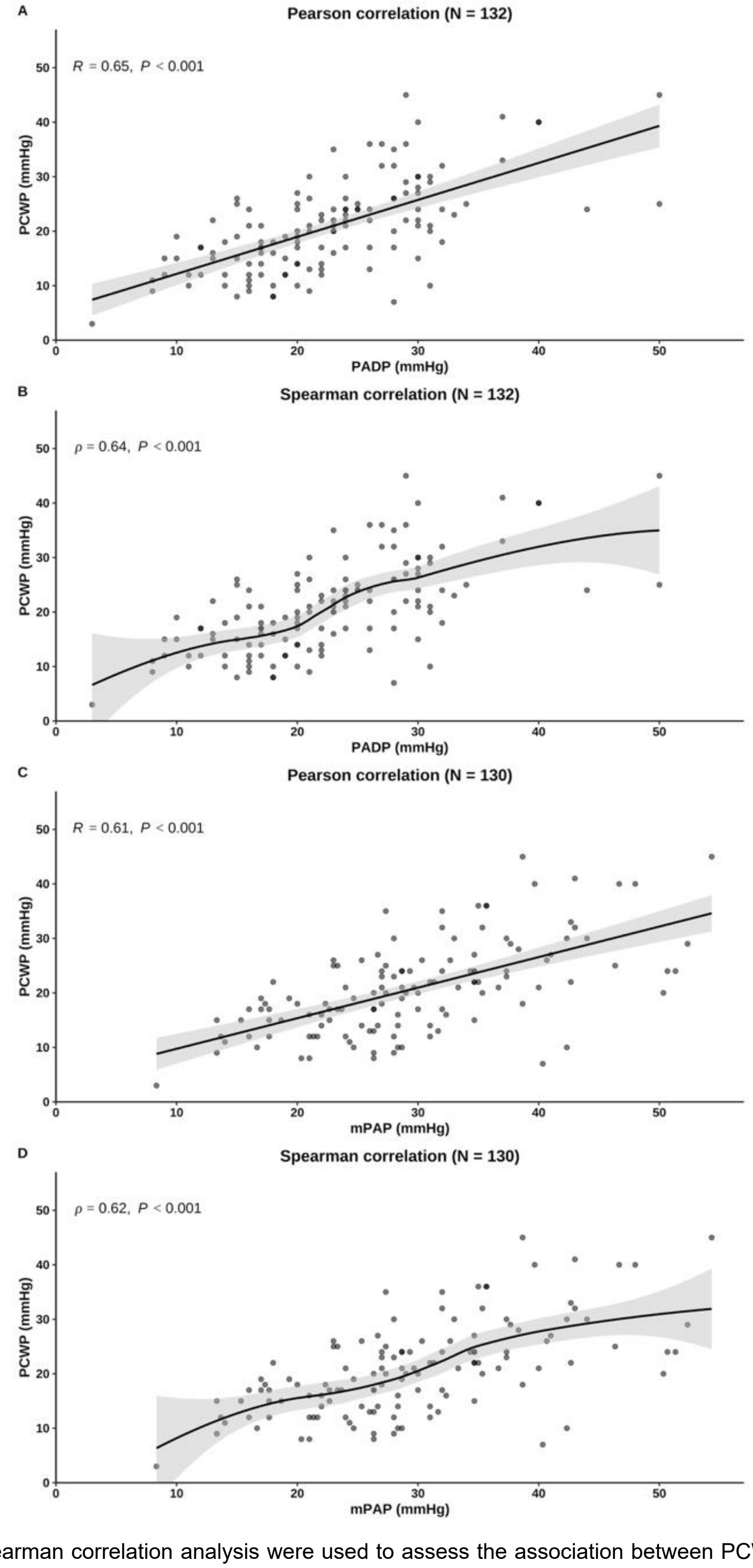

Pearson and Spearman correlation analysis were used to assess the association between PCWP, PADP **(A, B**), and mPAP (**C, D**) in patients with acute myocardial infarction–related cardiogenic shock (AMI-CS). AMI-CS = acute myocardial infarction–related cardiogenic shock; PCWP = pulmonary capillary wedge pressure; PADP = pulmonary artery diastolic pressure; mPAP = mean pulmonary artery pressure.

Figure S3: Distribution of API and mAPI Across Baseline SCAI Shock Stages in AMI-CS.

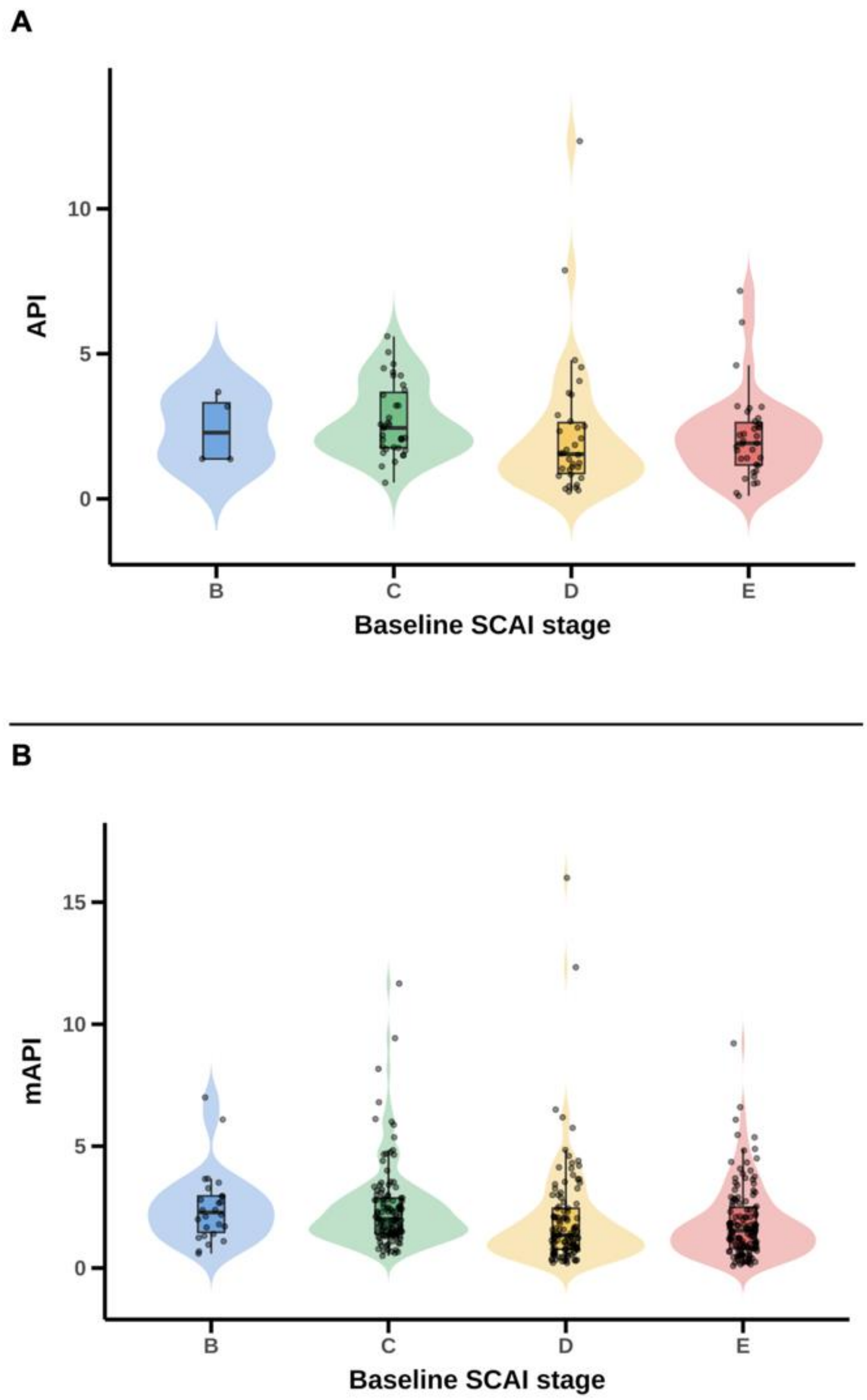

(A) Distribution of Arterial Pulsatility Index (API) across baseline Society for Cardiovascular Angiography and Interventions (SCAI) shock stages B to E. **(B)** Corresponding distribution of Modified API (mAPI) across SCAI stages. Dots represent individual patient values, violin plots showing median, interquartile range, and kernel density.

Figure S4: Association Between API/mAPI and Left Ventricular Ejection Fraction in AMI-CS.

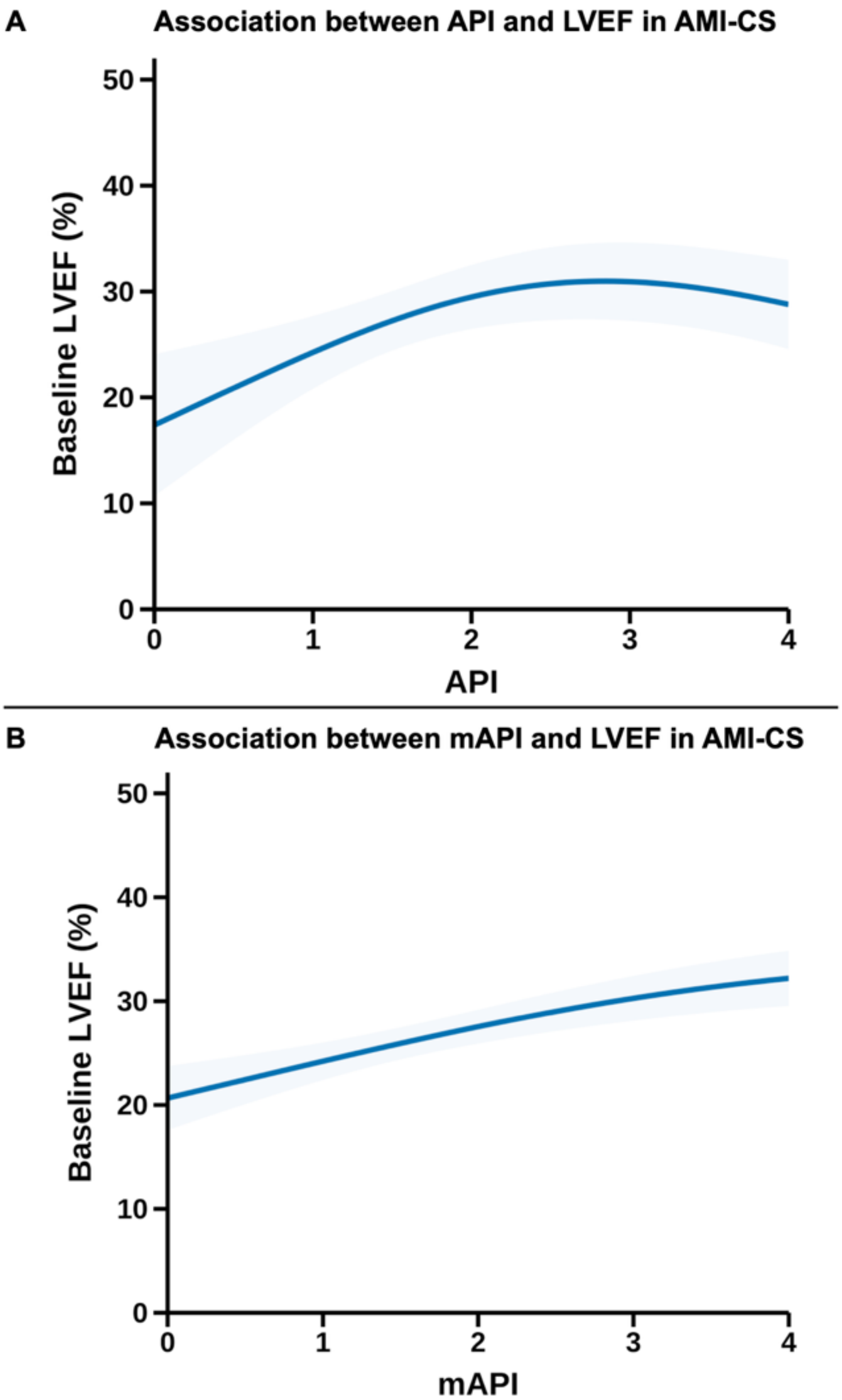

Generalized additive models with cubic regression splines were used to evaluate the association between the Arterial Pulsatility Index (API) and modified API (mAPI) with baseline left ventricular ejection fraction (LVEF) in patients with AMI-related cardiogenic shock. **(A)** Association between API and LVEF. **(B)** Association between mAPI and LVEF. Blue lines represent estimated LVEF across the continuous range of API/mAPI values; shaded areas indicate 95% confidence intervals. AMI-CS = acute myocardial infarction–related cardiogenic shock; API = arterial pulsatility index; LVEF = left ventricular ejection fraction; mAPI = modified arterial pulsatility index.

Figure S5: Crude In-Hospital Mortality Across SCAI Shock Stages Stratified by API and mAPI.

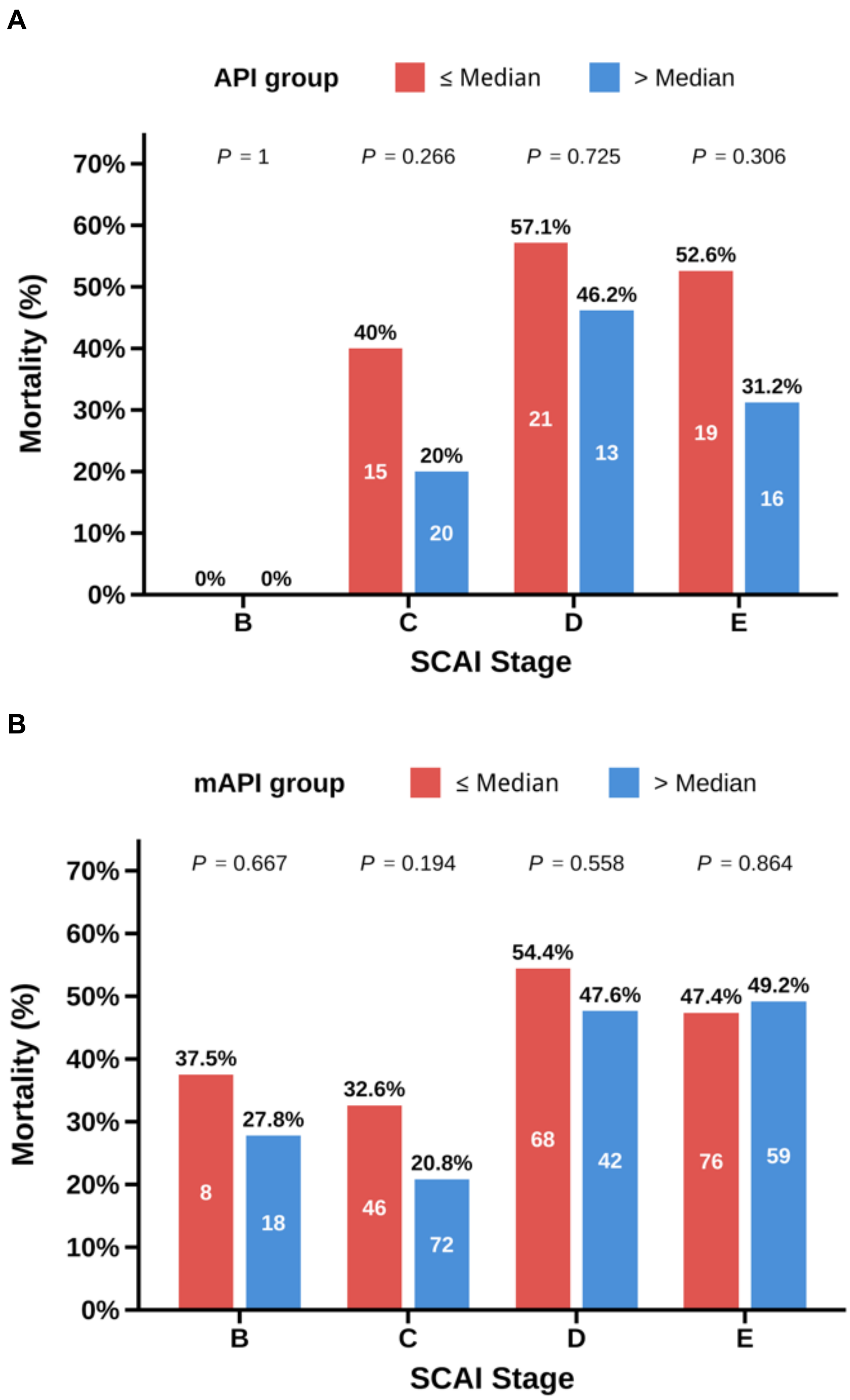

(A) Crude in-hospital mortality across baseline Society for Cardiovascular Angiography and Interventions (SCAI) shock stages, stratified by median Arterial Pulsatility Index (API; ≤ vs. > median). **(B)** Corresponding mortality rates stratified by median modified API (mAPI). P values were calculated using chi-square tests.

***Figure S6**:* Predicted Risk of In-Hospital Mortality by API and mAPI in AMI-CS.

A

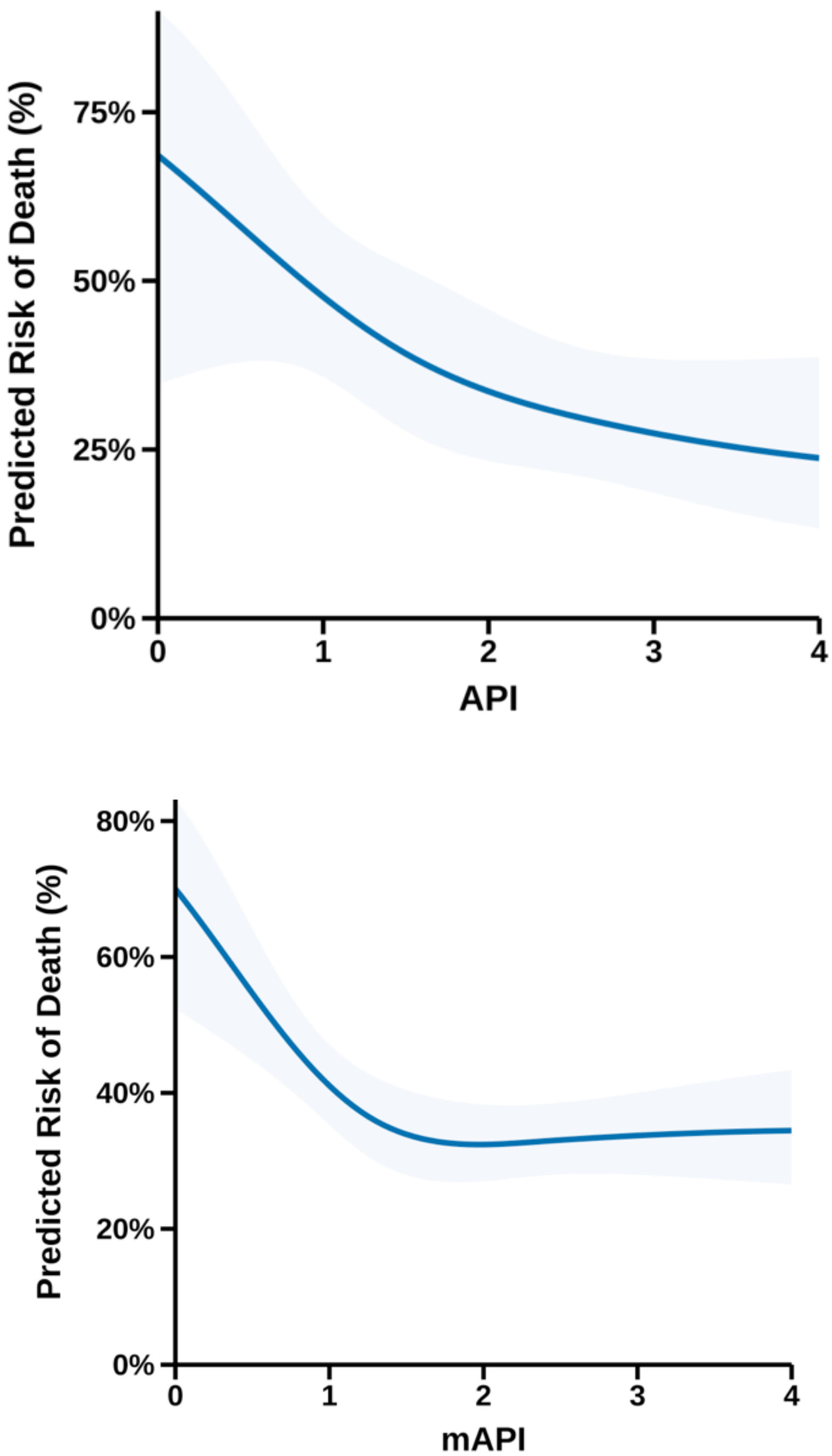

(A) Predicted risk of in-hospital mortality across the Arterial Pulsatility Index (API), using restricted cubic spline models, based on logistic regressions. **(B)** Corresponding predicted in-hospital mortality risk across modified API (mAPI). Blue lines indicate estimated risk, and shaded areas represent 95% confidence intervals.

***Figure S7**:* Discriminative Performance of API/mAPI and their Individual Hemodynamic Components.

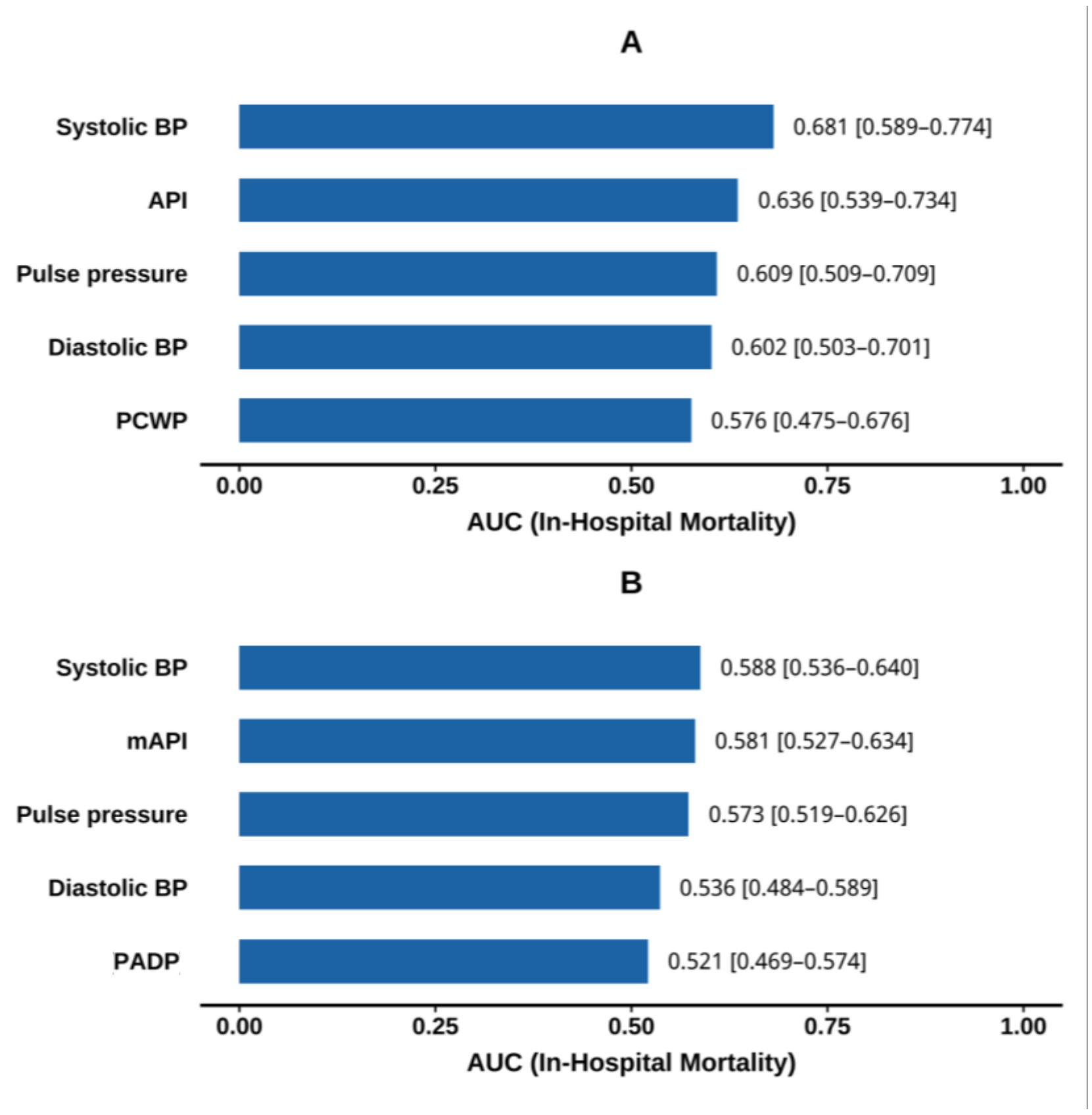

(A) API and individual hemodynamic measures. **(B)** mAPi and individual hemodynamic measures. API = arterial pulsatility index; mAPI = modified arterial pulsatility index; PCWP = pulmonary capillary wedge pressure; PADP = pulmonary artery diastolic pressure; AUC = area under the receiver operating characteristic curve.
